# Beitrag des BfArM zur Potenzialentfaltung der Digitalisierung im Gesundheitswesen – digital readiness@BfArM

**DOI:** 10.1007/s00103-021-03417-7

**Published:** 2021-09-15

**Authors:** Karl Broich, Wiebke Löbker, Wolfgang Lauer

**Affiliations:** Bundesinstitut für Arzneimittel und Medizinprodukte (BfArM), Kurt-Georg-Kiesinger-Allee 3-5, 53175 Bonn, Deutschland

**Keywords:** BfArM, Big Data, Forschungsdatenzentrum, Interoperabilität, Künstliche Intelligenz, DiPA, BfArM, Big data, Research data center, Interoperability, Artificial intelligence, DiPA

## Abstract

Die Digitalisierung stellt auch im Gesundheitsbereich einen eindeutigen Megatrend dar, der neben der Wegbereitung durch geänderte rechtliche Rahmenbedingungen auch durch die Coronapandemie eine enorme Beschleunigung erfahren hat. Mit Blick in die Zukunft wird dieser Trend durch immer kürzere Entwicklungszyklen und technologischen Fortschritt zu einer zunehmenden Verschmelzung einzelner digitaler und nicht digitaler Produkte, darunter Arzneimittel und digitale Medizinprodukte, zu einem digitalen Ökosystem beitragen. Die Digitalisierung wird nicht nur die Patientensouveränität stärken, sondern unter anderem auch eine patientenzentrierte, stärker auf den individuellen Patienten ausgerichtete Medizin ermöglichen; digitale (patientenberichtete) Endpunkte und „dezentrale“ Studienkonzepte werden die Möglichkeiten klinischer Studien erweitern. Künstliche Intelligenz wird Diagnosestellungen verbessern und beschleunigen und zum besseren Verständnis von Krankheitsbildern beitragen.

Um auch zukünftig Innovationen zu ermöglichen, aufkommende Trends im Fokus zu haben und vor allem die Patientensicherheit weiter zu verbessern, trägt das Bundesinstitut für Arzneimittel und Medizinprodukte (BfArM) an vielen Stellen dazu bei, die mit der Digitalisierung verbundenen Chancen in Möglichkeiten zu verwandeln – ohne dabei die Risiken aus dem Blick zu verlieren: Dazu gehören der Ausbau des Forschungsdatenzentrums, Aufgaben zu Interoperabilität und Klassifikationen sowie Ansätze zur datengestützten Signalerkennung aus Risikoinformationen. Wie darüber hinaus Forschungsprojekte unter Nutzung von künstlicher Intelligenz, (inter)nationale Kooperationen, die Einbeziehung von Real World Data in Nutzen-Risiko-Bewertungen, die Bewertung digitaler Gesundheits- und Pflegeanwendungen neben weiteren Aktivitäten zur „digital readiness“ in Deutschland und Europa beitragen, dazu im Folgenden ein Überblick.

## Abstract (en)

Digitalization is a clear megatrend of our time, also in the health sector, which is currently experiencing enormous acceleration due to the COVID-19 pandemic in addition to paving the way due to changes in the legal framework. Looking to the future, this trend will contribute to further digitalization and the merging of individual digital products, including medicinal products and medical devices, into a digital ecosystem. This will be supported by ever-shorter development cycles and technological progress. Digitization will not only strengthen patient sovereignty, but also enable more patient-centered medicine; artificial intelligence will improve and accelerate diagnoses and will contribute to a better understanding of disease patterns and underlying mechanisms or causes.

In order to continue to enable innovations in the future, to focus on emerging trends, and, above all, to further improve patient safety, the BfArM is contributing in many places to transforming the opportunities associated with digitalization into possibilities – without losing sight of the risks. The following is an overview of how, for example, the expansion of the Research Data Center, activities addressing interoperability, research projects using artificial intelligence, (inter-)national cooperation, the utilization and inclusion of “Real World Data” in our benefit/risk assessments, and the evaluation of digital health and digital care applications among other activities of the BfArM contribute to “digital readiness” in Germany and Europe.

## Einleitung

Wie sieht die Zukunft im Gesundheitsbereich aus? Soviel lässt sich an dieser Stelle schon sagen: Sie wird auf jeden Fall sehr viel digitaler. Mit der Digitalisierung werden viele Chancen für eine verbesserte, effizientere Versorgung, vor allem auch in infrastrukturschwachen Regionen, sowie für die Entwicklung neuer Therapieoptionen verbunden. Die sinnvolle Verknüpfung von Daten ermöglicht die Verbesserung präventiver, diagnostischer und medizinisch-therapeutischer Maßnahmen. Immer mehr digitale Angebote, die z. B. therapiebegleitend Daten erfassen, sammeln, zusammenführen und auswerten, durchdringen daher den Gesundheitsmarkt und erfahren in Form von Apps, Wearables, Plattformtechnologien oder telemedizinischen Ansätzen zunehmenden Zuspruch [1, 2].

Die nationale E‑Health-Strategie der Bundesregierung sowie geänderte gesetzliche Rahmenbedingungen haben in den letzten Jahren den Weg für eine stärkere Digitalisierung im Gesundheitsbereich geebnet, um Weichen für eine bessere Gesundheitsversorgung zu stellen: So haben, um einige aktuelle Beispiele zu nennen, durch das Digitale-Versorgung-Gesetz (DVG) digitale Gesundheitsanwendungen (DiGA), den Einzug in die Regelversorgung der gesetzlichen Krankenversicherung (GKV) gefunden und mit dem Terminservice- und Versorgungsgesetz sind gesetzliche Krankenkassen dazu verpflichtet, ihren Versicherten die elektronische Patientenakte (ePA) anzubieten. Das elektronische Rezept wird mit dem Gesetz für mehr Sicherheit in der Arzneimittelversorgung (GSAV) sukzessive eingeführt. Auf Basis des Patientendaten-Schutz-Gesetzes (PDSG) werden mit der Einführung der Terminologie SNOMED CT in Deutschland und somit durch eine Stärkung der semantischen Interoperabilität wichtige Voraussetzungen für eine einheitliche und sinnvolle Nutzbarmachung der – exponentiell zunehmenden – Gesundheitsdaten, vor allem auch aus dem „realen Versorgungsalltag“ (Real World Data) in Deutschland und Europa geschaffen.

Mit Projekten wie der Medizininformatikinitiative [3], „GAIA X“ [4] oder dem „Data Analytics and Real World Interrogation Network (DARWIN EU)“ [5] werden wichtige Dateninfrastrukturen als Wegbereiter für den geplanten Europäischen Gesundheitsdatenraum (EHDS; [6]) geschaffen. Damit wird gleichzeitig eine Brücke zwischen Forschungsfragen und praktischer, alltäglicher medizinischer Gesundheitsversorgung gebaut. Standardisierte medizinische Inhalte und Daten sind wichtige Grundlagen für diese Bereiche sowie für regulatorische Fragestellungen zu Nutzen und Risiken der auf dem Markt befindlichen Arzneimittel und Medizinprodukte gleichermaßen und stellen eine wichtige Voraussetzung für die interoperable Vernetzung einer digitalen Infrastruktur im Zusammenspiel aus DiGA, ePA, Telematikinfrastruktur (TI) etc. dar.

Neben dem wichtigen Aspekt der Standardisierung stellt auch der Ausbau der bisherigen Datentransparenzstelle durch das DVG und die Änderung der Datentransparenzverordnung zu einem Forschungsdatenzentrum beim Bundesinstitut für Arzneimittel und Medizinprodukte (BfArM) eine wichtige Grundlage für eine zeitnahe und zielführende Bereitstellung und Analyse der Gesundheitsdaten von ca. 70 Mio. gesetzlich Versicherten dar.

Unter Nutzung von Methoden der künstlichen Intelligenz (KI) erschließen sich ganz neue Möglichkeiten für diagnostische und therapeutische Fragestellungen sowie für eine schnellere und effizientere Risikosignalerkennung und -bewertung.

**Figure 1:**
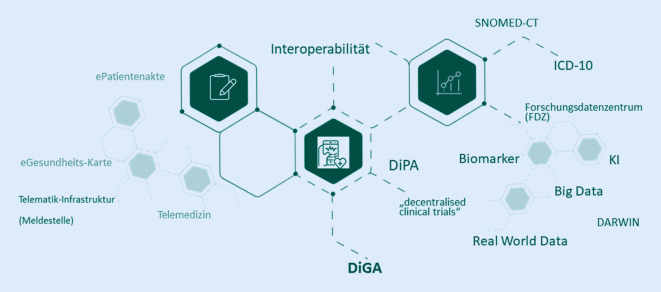

Das BfArM nimmt also im Zusammenwachsen und Zusammenspiel der einzelnen digitalen Komponenten (Abb. 1) einer modernen Gesundheitsversorgung schon jetzt Aufgaben an zentralen Schnittstellen für die (digitalgestützte) Patientenversorgung der Zukunft wahr und stellt sich dabei innovativen Ansätzen – unter Wahrung der für die Nutzer wichtigen Aspekte wie Datenschutz und Informationssicherheit, die sich u. a. aus der Datenschutz-Grundverordnung oder dem DVG ergeben.

Gleichzeitig stellen die zunehmend zu beobachtende Vielfalt, Komplexität und Dynamik digitaler Produkte, immer kürzere Entwicklungszyklen, das stärkere Zusammenwachsen von Arzneimitteln und Medizinprodukten im Versorgungsalltag, dezentralisierte Studienansätze und die zunehmenden Datensätze nicht nur Chancen für eine patientenzentrierte, bessere und zunehmend diesbezüglich integrierte Gesundheitsversorgung dar, sondern bringen auch neue Herausforderungen und Risiken – wie zu Datenqualität, Datenschutz, Cybersicherheit, Interoperabilität, ethischen Fragestellungen u. a. – mit sich.

Mit seinen zentralen Aufgaben, aber auch mit einer engen Zusammenarbeit auf nationaler und europäischer Ebene setzt sich das BfArM proaktiv dafür ein, die Chancen einer digitalisierten Gesundheitsversorgung in patientenrelevante Möglichkeiten mit Mehrwert für die Versorgung der Patientinnen und Patienten zu verwandeln. Dazu gehört auch, die damit verbundenen Herausforderungen gezielt anzugehen, mit den dynamischen Entwicklungen auf einer Höhe zu sein und bestehende Prozesse und Verfahren, wie zum Beispiel den DiGA-Fast-Track oder die Risikobewertung bei Arzneimitteln und Medizinprodukten, kontinuierlich weiterzuentwickeln, digitale Innovationen auf ihrem Weg zu wirksamen und sicheren Produkten durch Beratungs- und Informationsangebote zu begleiten und zu fördern oder beispielsweise Rahmenbedingungen für die Nutzung großer Datensätze (Big Data) bzw. Real World Data zu schaffen.

Der vorliegende Beitrag gibt einen Überblick über die Aufgaben des BfArM im Zusammenhang mit der Digitalisierung des Gesundheitswesens und beleuchtet die jeweiligen Hintergründe.

### Big Data/Real World Data: Neue Datenquellen für Nutzen-Risiko-Bewertungen

Die wissenschaftliche Bewertung von Daten bildet die Grundlage für die Nutzen-Risiko-Entscheidungen der Zulassungs- und Risikobewertungsbehörden. Diese Daten stammten im Rahmen von z. B. Zulassungsanträgen bei Arzneimitteln bisher überwiegend aus randomisierten kontrollierten klinischen Studien. Aufgrund ihrer hohen internen Validität gelten diese auch immer noch als Goldstandard, die Ergebnisse lassen sich im Versorgungsalltag aber aufgrund der niedrigeren externen Validität nicht immer so bestätigen. Die Weiterentwicklung und Nutzung zusätzlicher Datenquellen, z. B. aus digitalen Anwendungen im Versorgungsalltag, aus Berichten von Ärzten und Anwendern zu unerwünschten Arzneimittelwirkungen und Vorkommnismeldungen bei Medizinprodukten oder auch von Registerdaten, liefern hier wichtige komplementäre Informationen. Auch wenn diese Real-World-Evidenzdaten dadurch zunehmend vielfältiger und heterogener werden, ermöglichen solche Ansätze eine bessere Berücksichtigung der Versorgungsrealität. Daten aus Registern, DiGA, Wearables etc. können gerade bei seltenen Erkrankungen oder bei Fragen zum kontinuierlichen Therapie‑/Krankheitsverlauf wichtige zusätzliche Informationen liefern.

Um diese Daten sinnvoll und strukturiert zu nutzen und in die Evaluation von Nutzen und Risiken vor der Zulassung von Arzneimitteln, aber auch im gesamten Lebenszyklus im Rahmen der Vigilanzaufgaben zu integrieren, setzt sich das BfArM mit wichtigen Fragen in diesem Kontext auseinander: Welche Datenquellen haben einen Mehrwert und (wie) können andere (zum Teil neue) Datenformen bei der Nutzen-Risiko-Bewertung einbezogen werden? Welcher Auswertungsstrategien bzw. -methoden bedarf es, um diesen umfangreichen Datensatz, der über die Daten aus den „klassischen Studien“ hinausgeht, zu bewerten und welche Anforderungen stellt das an unsere Infrastruktur und die erforderliche Expertise? Welcher Standards – semantischer und technischer Interoperabilität – bedarf es, damit alle im Kontext dieser Daten „die gleiche Sprache“ sprechen?

So beforscht das BfArM zum Beispiel innovative Ansätze zur datengestützten Risikosignalerkennung und -bewertung bei Medizinprodukten und überführt diese in die regulatorische Praxis. Im Rahmen seiner Risikobewertung zu Vorkommnismeldungen steht das BfArM dabei vor der Herausforderung, schnell und zuverlässig übergreifende Ursachenzusammenhänge und Risikomuster bei zunehmend komplexen Medizinprodukten zu erkennen und zu bewerten sowie ggf. erforderliche Maßnahmen zur Risikominimierung abzuleiten. Hinzu kommt die praktische Herausforderung, dass die Anzahl der gemeldeten Vorkommnisse seit Jahren exponentiell ansteigt und inzwischen bei über 20.000 Meldungen pro Jahr liegt, die entsprechend bewertet werden müssen. Das BfArM entwickelt daher in seiner Forschungsgruppe zur Medizinproduktesicherheit wissenschaftliche Ansätze und praktische Analysetools z. B. auf Basis von KI. Diese können die Assessorinnen und Assessoren in der regulatorischen Praxis bei der schnellen Bewertung eingehender Meldungen unterstützen, indem sie z. B. wichtige Hinweise zu vergleichbaren Fällen geben oder einen erweiterten, auch produkt- und herstellerübergreifenden Überblick zu Risikomustern und Ursachenverläufen ermöglichen. Diese Ansätze werden in Zukunft weiter ausgebaut und z. B. hinsichtlich neuer, stärker trend- und signalbezogener Risikobewertungsverfahren ergänzt.

Daten stammen aber nicht nur aus nationalen Quellen, sondern werden heutzutage global generiert; das BfArM bringt daher seine Expertise in die europäischen Gremien und Arbeitsgruppen ein, wie zum Beispiel in die Big Data Steering Group, um gemeinsam Lösungsansätze zu erarbeiten, Standards zu definieren und so das Potenzial der großen Datenmengen zu erschließen.

### Künstliche Intelligenz und maschinelles Lernen – Anwendungsbeispiele im BfArM

Ansätze künstlicher Intelligenz (KI) entwickeln sich mit hoher Geschwindigkeit und Dynamik in vielen Lebensbereichen und werden z. B. auch zunehmend zum Bestandteil von Medizinprodukten. Neben vielen Chancen ergeben sich daraus auch neue regulatorische Herausforderungen z. B. zur Bewertung von Risiken „lernender Produkte“, die sich während der klinischen Anwendung ohne direkte Einflussnahme eines Menschen fortentwickeln. Dies betrifft z. B. Fragen der Prüf- und Validierungsmöglichkeiten (initial sowie im Anwendungsverlauf), Fragen der Verantwortlichkeit des Herstellers und der Anwender (die durch Anwendung den Lernprozess des Systems beeinflussen) und schließlich Cybersicherheitsrisiken, die sich z. B. aus Angriffen auf die Datenintegrität der Systeme ergeben können.

Das BfArM beschäftigt sich neben der Forschung zur eigenen Anwendung von KI im Rahmen seiner Risikobewertung auch intensiv mit Fragen zur Bewertung entsprechender Ansätze als Bestandteil von Medizinprodukten. Es beteiligt sich dazu aktiv an Forschungsprojekten, wie z. B. dem aktuellen Verbundprojekt „KIMEDS: KI-assistierte Zertifizierung medizinischer Software“ in der Ausschreibung des Bundesministeriums für Bildung und Forschung (BMBF) „Medizintechnische Lösungen für eine digitale Gesundheitsversorgung“, sowie an entsprechenden Arbeitsgruppen und europäischen wie internationalen Initiativen, wie z. B. der ITU/WHO^1^-Fokusgruppe AI4Health [8].

Auch im Arzneimittelbereich kommt im BfArM bereits KI zum Einsatz: So entwickelt das BfArM ein Modell des maschinellen Lernens, welches Meldungen zu Nebenwirkungen automatisch bewerten sowie kausale Zusammenhänge zwischen Arzneimittel und Nebenwirkung erkennen und klassifizieren soll. Und in pharmakologischen/genetischen Studien werden umfangreiche Datensätze (mit Einwilligung der Probanden) anschließend analysiert und statistisch ausgewertet, indem diese nach definierten Mustern durchsucht bzw. auf Assoziationen zwischen interessierenden Merkmalsausprägungen getestet werden.

### 5G-Standards für die Gesundheitsversorgung

Das BfArM arbeitet kontinuierlich an Möglichkeiten zur Verbesserung und Erweiterung seiner Risikobewertung bei Arzneimitteln und Medizinprodukten. So untersucht es z. B. derzeit im Rahmen eines durch das Bundesministerium für Gesundheit (BMG) geförderten Forschungsprojektes mögliche Hürden und Lösungsansätze zur Unterstützung des Risikomeldens bei der Anwendung von Medizinprodukten, da diese Meldungen die zentrale Säule der schnellen und zuverlässigen Risikosignalerkennung und -bewertung bilden. Darüber hinaus beteiligt sich das BfArM z. B. aktiv an dem aktuellen Forschungsprojekt „GIGA FOR HEALTH“ im Rahmen des Förderrahmens „5G.NRW“, in dem gemeinsam mit der Universitätsklinik Düsseldorf und vielen weiteren Partnern aus Forschung und Industrie neue Möglichkeiten zur Nutzung des 5G-Standards für die Gesundheitsversorgung erforscht werden. Das BfArM wird in diesem Zusammenhang insbesondere eine App zur schnellen, mobilen Erfassung und Meldung von Risiken bei Medizinprodukten entwickeln und gemeinsam mit den klinischen Partnern in der Praxis evaluieren. Durch die Nutzung des 5G-Standards können entsprechende Risikomeldungen einschließlich ggf. umfangreicher multimedialer Zusatzinformationen, wie z. B. Fotos und Filme, schneller und sicherer übertragen werden.

### Forschungsdatenzentrum

Auf Basis des DVG und der Neufassung der Datentransparenz-Verordnung (DaTraV) wird die bisherige Datentransparenzstelle beim ehemaligen Deutschen Institut für medizinische Dokumentation und Information (DIMDI) parallel zur Integration des DIMDI in das BfArM zu einem leistungsstärkeren Forschungsdatenzentrum ausgebaut. Dieses wird Nutzungsberechtigten im Vergleich zu den bisherigen Möglichkeiten einen umfassenderen, aktuelleren Datensatz auf Basis pseudonymisierter Daten der gesetzlichen Krankenkassen für wissenschaftliche Fragestellungen der Versorgungsforschung zur Verfügung stellen. Kritikpunkte am alten System und Nutzeranforderungen der modernen Versorgungsforschung werden dabei besonders berücksichtigt. Konkret wird durch die Schaffung einer Analyseplattform (unter Berücksichtigung des angebracht hohen Datenschutzes) ein iterativer Workflow mit kurzen Warte- und Bearbeitungszeiten ermöglicht.

### Semantische, syntaktische und technische Interoperabilität

Ein wesentlicher Erfolgsfaktor für die gesamte Digitalisierungsstrategie ist die Verwendung einer gemeinsamen Terminologie sowie kompatibler technischer Standards. Ohne diese Interoperabilität ist eine Zusammenführung großer Datenmengen, eine sinnvolle Analyse dieser Daten (z. B. für longitudinale Studien oder sektorenübergreifende Fragestellungen) oder eine Auswertung für Abrechnungszwecke nicht möglich. Mit Blick auf das Zusammenspiel mit der ePA, IT-Systemen in den Arztpraxen etc. ist beispielweise Interoperabilität eine Anforderung an DiGA, die für die Aufnahme in die Regelversorgung zu erfüllen ist, damit eine entsprechende Weiterverarbeitung der Daten aus den DiGA möglich ist.

Als nationales Kompetenzzentrum für medizinische Terminologien und Klassifikationen stellt das BfArM mit dem ICD-10-Diagnoseschlüssel sowie mit SNOMED CT Terminologiestandards [9] bereit und schreibt diese fort bzw. trägt aktiv zu deren Weiterentwicklung bei. Zudem wird aufgrund der zunehmenden Bedeutung aktuell eine Plattform für semantische Standardisierungswerkzeuge (Semantikzentrum) ausgebaut, in Zusammenarbeit mit den relevanten Organisationen des Gesundheitswesens [10].

Zudem hat das BfArM mit dem Digitale Versorgung und Pflege – Modernisierungs-Gesetz (DVPMG) u. a. die neue Aufgabe erhalten, ein elektronisches Verzeichnis für interoperable Schnittstellen von solchen Hilfsmitteln und Implantaten aufzubauen und weiterzuführen, die zulasten der GKV an Versicherte abgegeben werden [11]. Darin werden die auf Basis einer entsprechenden Festlegung des BfArM von den Herstellern gemeldeten interoperablen Schnittstellen der jeweiligen Produkte veröffentlicht. So wird sichergestellt, dass im DiGA-Verzeichnis des BfArM gelistete digitale Gesundheitsanwendungen die von einem Implantat oder sonstigen Hilfsmittel generierten Daten nach entsprechender Einwilligung der Nutzer zum bestimmungsgemäßen Gebrauch der DiGA durch denselben Versicherten verarbeitet werden können.

Auf Basis der vielfältigen bisherigen Erfahrungen mit dem DiGA-Fast-Track-Verfahren des BfArM sieht das DVPMG ebenfalls eine Erweiterung hin zu digitalen Pflegeanwendungen (DiPA) vor, die in Abgrenzung zu den DiGA neben der technischen Sicherheit und Leistungsfähigkeit sowie Nachweisen zu Datenschutz und Datensicherheit insbesondere einen pflegerischen Nutzen, also eine unmittelbare Unterstützung des pflegerischen Handelns bieten sollen. Auch hier wird das BfArM gemeinsam mit dem BMG ein entsprechendes Bewertungsverfahren sowie ein transparentes und umfassend nutzbares elektronisches Verzeichnis entsprechender Anwendungen aufbauen und veröffentlichen.

Damit begrüßt das BfArM die Erweiterung der bisherigen innovativen Schritte zur Etablierung digitaler medizinischer Anwendungen in der Gesundheitsversorgung und deren diesbezügliche Unterstützung im Hinblick auf die Kostenerstattung durch die gesetzliche Kranken- bzw. Pflegeversicherung.

### Datenschutz und Informationssicherheit durch Zertifikate stärken

Eine weitere Entwicklung, die der Gesetzgeber durch das DVPMG vorgenommen hat, ist – basierend auf den ersten Erfahrungen im DiGA-Fast-Track – eine Anpassung der Checklisten zu Datenschutz und Informationssicherheit, Interoperabilität und Barrierefreiheit der Digitale-Gesundheitsanwendungen-Verordnung für die bisherige Selbstauskunft der Hersteller. Hier erarbeiten das BfArM und das BMG zusammen mit u. a. dem Bundesbeauftragten für den Datenschutz und die Informationsfreiheit (BfDI) sowie dem Bundesamt für Sicherheit in der Informationstechnik (BSI) Zertifikate, die alle wesentlichen Aspekte und Anforderungen an Datenschutz und Informationssicherheit, ganz konkret auf die Eigenschaften der DiGA (bzw. zukünftig auch DiPA) zugeschnitten, abbilden und damit zukünftig als Prüfnachweis für entsprechende Anforderungen im Rahmen des DiGA-Bewertungsprozesses dienen werden. Somit werden eine höhere Transparenz und Klarheit zu den Anforderungen an Datenschutz und Informationssicherheit geschaffen.

### Aufkommende Trends frühzeitig in den Blick nehmen

Neben dem konstruktiven Dialog mit Entwicklern und Herstellern etabliert das BfArM einen systematischen „Horizon-Scanning“-Ansatz, aufkommende wissenschaftliche wie technologische Trends frühzeitig zu identifizieren, deren Einfluss auf das regulatorische Umfeld zu analysieren, um so Prozesse und Informationen frühzeitig anpassen und Expertise aufbauen und bereitstellen zu können.

### Beratungs- und Unterstützungsangebote

Das BfArM hat den „digitalen Trend“ durch den intensiven Austausch mit Herstellern frühzeitig erkannt und leistet mit diversen Unterstützungs- und Beratungsangeboten Hilfestellung zu regulatorischen Rahmenbedingungen, damit Patientinnen und Patienten unverzögerten Zugang zu (digitalen) Innovationen erhalten. Bereits 2014 hat das BfArM eine Orientierungshilfe zur Einordnung von Apps als Medizinprodukt veröffentlicht [12] und seitdem in mehreren „BfArM-im-Dialog“-Veranstaltungen mit Entwicklern, Aufsichtsbehörden, aber auch Hackern zentrale Aspekte medizinprodukterechtlicher Themen bis hin zu Cybersicherheitsaspekten aus unterschiedlichsten Perspektiven diskutiert. 2017 wurde mit dem Innovationsbüro [13] eine erste Anlaufstelle insbesondere für Start-ups, akademische Forschungseinrichtungen und kleine bzw. mittelständische Unternehmen eingerichtet, um diesen in frühen Entwicklungsphasen Orientierung auf dem Weg zum Marktzugang ihrer (digitalen) innovativen Ansätze zu geben. In diesem Kontext hat das BfArM unter anderem auch im Vorfeld des Inkrafttretens des DVG im Rahmen einer „Roadshow“ gemeinsam mit BMG und dem health innovation hub (hih) des BMG über die Möglichkeiten des DVG und entsprechende Beratungsangebote des BfArM informiert [14].

### Kooperationen und Projekte auf nationaler und europäischer Ebene

Digitalisierung kann nur in der Gesamtschau der einzelnen Digitalbausteine und Schnittstellen sowie relevanter Aspekte insbesondere aus dem medizinischen, informationstechnischen, arzneimittel-, medizinprodukte- und sozialrechtlichen Kontext zum Erfolg werden. Daher arbeitet das BfArM eng mit weiteren Akteuren zusammen. Dazu gehören auf nationaler Ebene insbesondere die gematik GmbH, das Bundesministerium für Gesundheit, der health innovation hub des Bundesgesundheitsministeriums, der Bundesbeauftragte für den Datenschutz und die Informationsfreiheit (BfDI), das Bundesamt für Sicherheit in der Informationstechnik (BSI) sowie zahlreiche Kooperationen z. B. mit akademischen Forschungseinrichtungen.

Auf europäischer Ebene ist das BfArM unter anderem mit mehreren Vertretern aktiv in die „HMA/EMA Big Data Steering Group“ eingebunden, um auch hier im interdisziplinären Kontext eine umfassende Bewertung der Herausforderungen und Chancen von Big Data für die regulatorischen (Nutzen-Risiko‑)Bewertungen zu diskutieren und entsprechende Empfehlungen zu deren Nutzung umzusetzen, darunter als erste Priorität der Aufbau eines Data Analytics and Real World Interrogation Network (DARWIN EU), das als Wegbereiterinitiative und Anwendungsfall für den vorgeschlagenen europäischen Gesundheitsdatenraum (EHDS) dienen soll [6].

## Fazit

Die Digitalisierung verändert auch das Gesundheitswesen radikal und mit rasanter Geschwindigkeit, sie stellt eindeutig nicht nur einen aktuellen, sondern auch einen zukünftigen Trend mit weiteren vielfältigen Chancen für eine moderne, patientenzentrierte und effektive Gesundheitsversorgung dar. Mit dem Thema Digitalisierung verbindet das BfArM neben seinen gesetzlichen Aufgaben auch eine konkrete Vision [7] für ein digitales, interoperables Gesundheitsökosystem, an dessen konkreter Weiterentwicklung sich das BfArM aktiv mit den folgenden Aktivitäten und Themen einbringt – für eine innovative, effiziente Gesundheitsversorgung und insbesondere für mehr Patientensicherheit. Das BfArM gestaltet die digitale Transformation aktiv mit und trägt dazu bei, den deutschen Gesundheitsbereich bestmöglich auf die digitale Zukunft vorbereiten. Dazu gehört auch, neben den Chancen, welche eine zunehmende Digitalisierung für die Patientenversorgung unter Berücksichtigung des demografischen Wandels, zunehmende chronische Erkrankungen und steigende Kosten für innovative Therapien sowie wachsende, sinnvoll zusammengeführte Gesundheitsdaten bieten, die Herausforderungen, wie zum Beispiel Datenschutz und Informationssicherheit, zu meistern. Digitalisierung kann auch nicht isoliert betrachtet werden, sondern muss im interoperablen Zusammenspiel der einzelnen Digitalbausteine wie DiGA, DiPA, ePA, Telematikinfrastruktur etc. gedacht und gemeinsam in interdisziplinären Teams und Projekten gestaltet werden. Dazu nimmt das BfArM mit den hier beschriebenen aktuellen Aufgaben und Aktivitäten, aber auch der prospektiven Herangehensweise an aufkommende Trends und dem innovationsoffenen Mindset eine wichtige Schnittstelle ein. In Zusammenarbeit mit weiteren Akteuren des digitalen Gesundheitswesens auf nationaler wie auf europäischer Ebene leistet das BfArM einen wesentlichen Beitrag, damit eine digitale Gesundheitsversorgung, auch bereits unter Berücksichtigung zukünftiger digitaler Angebote und Trends, mit spürbarem Mehrwert den Bürgerinnen und Bürgern zugutekommt.

## Footnotes

1 ITU/WHO Focus Group on artificial intelligence for health (FG-AI4H); ITU: International Telecommunication Union; WHO: World Health Organization.
